# CAREGIVER BURDEN, BENEFIT, AND PERCEPTIONS OF END-OF-LIFE CARE QUALITY

**DOI:** 10.1093/geroni/igz038.2472

**Published:** 2019-11-08

**Authors:** Elizabeth A Luth, Teja Pristavec

**Affiliations:** 1 Weill Cornell Medicine, New York, New York, United States; 2 University of Virginia, Biocomplexity Institute and Initiative, Arlington, Virginia, United States

## Abstract

End-of-life care quality (EOLCQ) gauges our success in providing quality care to dying individuals. EOLQC measures rely on reports from bereaved family members who provide care for dying loved ones, but analyses seldom account for how caregivers’ experiences influence their EOLCQ perceptions. Caregivers frequently experience burden, which is linked to poor health outcomes and may negatively bias EOLCQ reports. Individuals may also perceive caregiving benefits that can offset deleterious burden effects, but potentially encourage overly positive EOLCQ perceptions. This paper links National Study of Caregivers (2011) and National Health and Aging Trends Study (2011-2016) data, using regression analysis and a sample of 380 EOL caregivers to examine how caregiving burden and benefits perceptions shape and moderate EOLCQ reports. Caregiving burden is unrelated to EOLCQ in adjusted models. Benefits are associated with marginally greater odds of being informed about the dying person’s condition and reporting their personal care needs were met. Burden and benefits moderate these two measures. Despite benefits, low burden caregivers report they were informed about the dying person’s condition with 90% probability. Regardless of burden, high benefits caregivers report the same with 90% probability. Low burden and benefits caregivers report met care needs with 90% probability. High burden and benefits caregivers have 90% probability of such reports. Given these reports are used in formal hospice care evaluations by CMS, additional research should explore why caregiving burden and benefit are associated with some EOLCQ measures and why individuals reporting high burden and benefits provide more positive EOLCQ appraisals.

